# Commissioning healthcare for people with long term conditions: the persistence of relational contracting in England’s NHS quasi-market

**DOI:** 10.1186/1472-6963-13-S1-S2

**Published:** 2013-05-24

**Authors:** Alison Porter, Nicholas Mays, Sara E Shaw, Rebecca Rosen, Judith Smith

**Affiliations:** 1 College of Medicine, Swansea University SA2 8PP, UK; 2 Professor of Health Policy, Department of Health Services Research and Policy, London School of Hygiene and Tropical Medicine, 15-17 Tavistock Place, London WC1H 9SH, UK; 3 Queen Mary University of London, Centre for Primary Care and Public Health, Blizard Institute, 8 Turner Street, London E1 2AB, UK; 4 The Nuffield Trust, 59 New Cavendish St, London W1G 7LP, UK

## Abstract

**Background:**

Since 1991, there has been a series of reforms of the English National Health Service (NHS) entailing an increasing separation between the commissioners of services and a widening range of public and independent sector providers able to compete for contracts to provide services to NHS patients. We examine the extent to which local commissioners had adopted a market-oriented (transactional) model of commissioning of care for people with long term conditions several years into the latest period of market-oriented reform. The paper also considers the factors that may have inhibited or supported market-oriented behaviour, including the presence of conditions conducive to a health care quasi-market.

**Methods:**

We studied the commissioning of services for people with three long term conditions - diabetes, stroke and dementia - in three English primary care trust (PCT) areas over two years (2010-12). We took a broadly ethnographic approach to understanding the day-to-day practice of commissioning. Data were collected through interviews, observation of meetings and from documents.

**Results:**

In contrast to a transactional, market-related approach organised around commissioner choice of provider and associated contracting, commissioning was largely relational, based on trust and collaboration with incumbent providers. There was limited sign of commissioners significantly challenging providers, changing providers, or decommissioning services.

In none of the service areas were all the conditions for a well functioning quasi-market in health care in place. Choice of provider was generally absent or limited; information on demand and resource requirements was highly imperfect; motivations were complex; and transaction costs uncertain, but likely to be high. It was difficult to divide care into neat units for contracting purposes. As a result, it is scarcely surprising that commissioning practice in relation to all six commissioning developments was dominated by a relational approach.

**Conclusions:**

Our findings challenge the notion of a strict separation of commissioners and providers, and instead demonstrate the adaptive persistence of relational commissioning based on continuity of provision, trust and interdependence between commissioners and providers, at least for services for people with long-term conditions.

## Introduction

Much research on commissioning in the National Health Service (NHS) examines the organisation of commissioning, and in particular, contracting (see paper by Hughes et al in this supplement [1]). Little attention has been paid to the day-to-day work of commissioning, including all the other tasks which support contracting, especially in relation to services for people with long term conditions. We therefore undertook a two year study (2010-2012) of the local commissioning of health care in three ‘commissioning communities’, each centred on an English NHS primary care trust (PCT). We focused on services for people with three long term conditions: diabetes, stroke and dementia [2]. The overall aim of the project was to explore how NHS commissioning could be enacted to improve care for people living with long-term conditions. The current analysis examines the extent to which commissioners had adopted a market-oriented (transactional) model of commissioning rather than the relational approach [1,3,4] that marked NHS community health services in the 1990s [5]. The paper also considers the factors that may have inhibited or supported market-oriented behaviour, including the presence of conditions conducive to a health care quasi-market.

### Commissioning in the English NHS quasi-market

Since 1991, the NHS in England has been organised around a separation between ‘commissioners’ (purchasers) and ‘providers’ of services. This separation is a defining characteristic of the so called NHS ‘quasi-market’ (‘quasi’ in that commissioners generally act on behalf of patients) in which public or independent providers of health care may compete for contracts from purchasers who are charged with securing services for their local population. Increasingly, too, there is scope for patient-initiated commissioning through choice of place of elective care and individual patient budgets. The theory is that competition (or the threat of competition) between suppliers of care will lead to more responsive, effective and efficient services than would occur if NHS services remained local monopolies (see the paper by Allen in this supplement for further detail [6]).

Following Williamson [3], Bartlett and Le Grand [7] posit a number of conditions that need to be satisfied for a quasi-market to function well in terms of efficiency, responsiveness, choice and equity (Table 1). This paper investigates the extent to which these were present at local level in relation to commissioning services for people with three long term conditions.

**Table 1:** Conditions which need to be satisfied for a quasi-market to work (adapted from Bartlett and Le Grand 1993)

competitiveness	the quasi-market should be competitive on both sides (many purchasers, many providers)
**information**	both providers and purchasers need access to accurate, independent information in order to monitor quality and minimise opportunistic behaviour on the part of the provider

**transaction costs**	(whether set-up or continuing) should be no higher than any cost savings generated by competition

**motivation**	economic theory suggest that providers should be motivated by financial considerations, and purchasers by user interest

**cream-skimming**	there should be no motivation for providers to pick and choose some patients over others on the base of cost or complexity of treatment

One of the most influential conceptualisations of health care commissioning, used by the English Department of Health to set out what is expected of NHS commissioners [8], distinguishes ‘commissioning’ from simple purchasing and/or contracting, suggesting that it has a more strategic and proactive intent [9]. This has been explained by Woodin as follows:

*‘A commissioner decides which services or health care interventions should be provided*, *who should provide them and how they should be paid for*, *and works closely with the provider implementing changes. A purchaser buys what is on offer or reimburses the provider on the basis of usage.’ *[10] (p203)

The framework for putting this intent into practice has been described in terms of an annual ‘commissioning cycle’, beginning with assessing the needs of the population, then planning services, contracting with providers, monitoring services and revising the previous plans, before embarking on another round of needs assessment [9].

This separation of commissioning from provision functions provides a structure which allows choice between alternative providers on the basis of cost and value, and the possibility of loss of contract to act as an encouragement to providers to maintain and improve the quality and appropriateness of services. However, there is no inherent connection between having a distinct commissioning function in a system and the existence of supplier competition, though the two are often confused in debate. Mays and Hand [11] identified various potential commissioning functions which do not necessarily depend on the operation of competition:

• a role in planning unhindered by responsibility for managing health care institutions or protecting employment of staff;

• acting as an informed counterweight to professional dominance of service specification and spending patterns;

• clarifying providers’ costs;

• facilitating clear lines of public accountability for the performance of providers and commissioners;

• making priority decisions more explicit to the public and patients.

### Policy context for the study

By the start of the research in March 2010, all the elements in the Labour Government’s quasi-market reforms [12,6] had been fully operational for about four years and PCTs had been in place for almost a decade. The majority of commissioning was carried out at local level, by 152 PCTs with responsibility for spending 75-80% of the NHS budget. Within the constraints of national strategy and targets, PCTs set local priorities, specified standards of quality for local services and could negotiate variations to the national standard service contracts, sometimes working on behalf of consortia of general practices under the ‘practice-based commissioning’ scheme. Though prices for most acute hospital services were fixed nationally based on Health Resource Groups (HRGs - the English equivalent of Diagnosis-Related Groups (DRGs)), other prices - including those for care delivered by community providers - were negotiated locally. Commissioners were also expected by the Department of Health to ‘stimulate’ the local health care market to ensure that ‘adequate provider choice’ existed [13].

The period of study was one of further major system change following the election of the coalition government in May 2010. The July 2010 NHS White Paper *Equity and Excellence: Liberating the NHS *[14] was the start of a tortuous legislative process leading towards a gradual shift of local responsibility for commissioning and for 60% of NHS resources to GP-led clinical commissioning groups (CCGs), fully implemented by April 2013. The other important contextual factor was a severe constraint on NHS budgets in a time of increasing demand [15].

## Methods

The study consisted of case studies of three ‘commissioning communities’, each centred on an English PCT, but also including other organisations involved in planning or delivering care. We studied the commissioning of health care for people with three long term conditions over 22 months (April 2010 and January 2012), taking a broadly ethnographic approach to understanding the day-to-day practice of commissioning.

A set of quantitative metrics, summarising 200 indicators drawn from the assessments by national agencies and local operational and financial data, was used to identify a group of ‘high performing’ PCTs which were invited to participate in the study. Somerset (pop 525,000), Wirral (pop 309,000) and Calderdale (pop 203,000) agreed to take part, with the Calderdale site subsequently expanded to include neighbouring Kirklees, reflecting local collaborative commissioning.

We studied six commissioning developments (two in each site). To allow comparison across sites, we examined an aspect of the commissioning of diabetes services in all three sites. Each PCT then selected a condition for which there was a local commissioning initiative: dementia in Calderdale and Wirral, and stroke in Somerset.

We gathered data through observing meetings (n=27), semi-structured interviews (n=124), informal update interviews (n=20) and analysis of documents (n=345). Observations were used to analyse the practice of commissioning. Fieldwork involved PCT managers and clinicians, general practice-based commissioners, NHS trust and NHS foundation trust senior managers and clinicians, and voluntary sector and local government representatives involved in planning and purchasing care.

There were three components to the analysis of this rich qualitative dataset, with the first two predominantly inductive. First, we developed a thematic framework from the data, informed by literature and guided by the approach of Hammersley, Gomm and Foster [16] that considered the processes and resources that appeared to shape commissioning outputs. The five key processes (driving change forward; addressing local people’s needs; specifying services and agreeing contracts; measuring and promoting service quality; and completing the commissioning cycle with review) and five resources (people/organisations, data, money, ideas and time) were used to create a matrix used as a working tool for analysis. Second, we wrote six working papers describing the commissioning practice in the six service areas being tracked, which allowed us to then explore themes within the data, and to compare practice within and across the sites. The on-going process of updating, review and reworking both elements of the analysis was made richer through involvement of all members of the multi-disciplinary research team, input from an external advisory group, and regular checking of our emerging analysis with the sites. The third element of our analysis, for the current paper, was deductive, using data from the first two elements to see whether the conditions for successful quasi-market relations (Table 1) were in place locally.

The study was approved by the Outer South-East London NHS Research Ethics Committee and local NHS Research Governance approval was gained in each site. We agreed to reveal the names of each of our three sites, but not identify individual respondents, so quotations presented below are anonymised.

## Results

We identify the extent to which each of Bartlett and Le Grand’s [7] five conditions for a successful quasi-market were in place (Table 1) by analysing the features of the commissioning activity in each site (summarised in Additional file 1). Finally, we present a sixth theme, not explicitly identified by Bartlett and Le Grand: the need to ‘parcel up’ service delivery into units for which specifications can be written and contracts issued by commissioners.

### Competitiveness

According to Bartlett and Le Grand [7], an effective quasi-market needs to be competitive on both sides; that is, have many purchasers, and many providers, or at least the scope for many providers to enter the market. Having single purchasers buying from single providers risks a ‘too intimate’ (p21) relationship that risks hampering the bargaining process. A market structure with many providers maintains quality and keeps costs down through the threat of loss of business offered by (potential) competition. Since each PCT was a monopoly purchaser within a geographical area for the services studied, we concentrate on the extent of competition among providers.

In only one of the six service areas did we observe de-commissioning of an old service occur as part of the process of service re-modelling. In Wirral, GPs ‘with a Special Interest’ (GPSIs) ceased to provide the diagnosis service for dementia patients when the new Wirral Memory Assessment Service was established (Additional file 1), delivered by the local NHS trust providing mental health care, with input from the Alzheimer’s Society. This shift in provider in Wirral did not take place through a formal process of competitive tendering and re-issue of contract. Instead, it was made through negotiation, a feature of commissioning that was common to all six service areas, with commissioners reluctant to destabilise the local health economy by suddenly withdrawing income from providers.

This approach to commissioning through negotiation with providers was seen very clearly in Somerset where, informed by a lengthy planning process, commissioners decided to procure the new intermediate level of diabetes care from the provider of community health services, rather than primary care provider. This was explained by a commissioning manager:

*‘There was a view that we would test a negotiated move or resource from secondary care to a community setting with [trust providing community health services] being the*, *the provider because they were the county wide provider of community services ... there are advantages in having a county wide provider and a county wide pathway as well.’*

Across the sites, the scope for new providers to enter the market was constrained by the limited supply of suitable specialist staff (Diabetes Specialist Nurses, for example, were in short supply nationally). While, in principle, a new provider could compete for available staff (offering them more attractive terms), in practice we observed that staff transfers and secondments became a lengthy part of the process of negotiation of service redesign. For example, the Somerset Early Supported Discharge (ESD) service was delivered by a range of specialist staff: occupational therapists, physiotherapists and speech and language therapists employed by the local NHS partnership trust (providing community health services in this area); specialist nurses employed by the acute trusts; and medical consultants from the acute trusts providing clinical oversight. There was some flow of staff between the partnership and acute trusts, largely via negotiated secondments. Managers and clinicians expressed a belief that it was important for providers, supported by commissioners, to co-operate rather than compete when it came to drawing on the pool of local staff in order to avoid disrupting other parts of provision. As one senior clinician commented when the ESD service was set up: *‘The danger would be that they would be robbing the existing stroke specialist services to start the new service.*’

### Information

In order for a quasi-market to operate efficiently, Bartlett and Le Grand [7] argue that commissioners and providers need access to cheap, accurate and independent information on cost and quality. This allows providers to set an appropriate price and purchasers to check that they are getting a good service. However, across all six service areas there were substantial challenges in relation to information, which can be grouped into two main types. The first was to do with the challenge of obtaining accurate information on all aspects of services, from activity data to information on quality and cost, to use in service redesign, with high expectations of performance and monitoring data rarely being met. In the Somerset ESD service, for example, a consistent theme of project meetings was the problem of getting accurate data about the numbers of patients going through ESD, and the numbers of those potentially eligible. The second set of challenges concerned incompatible and outdated information systems – including the continued reliance on paper records in some contexts - preventing the easy transfer of information between providers and from providers to commissioners. In Wirral, incompatible data systems meant that commissioners struggled to extract Read Code data from GP practices on the risk status of the feet of their diabetic patients. In another area, inadequate data systems meant that one senior clinician involved in reviewing a service area described resorting to going through records by hand:

‘I sat down for a weekend and actually went through a year’s worth of referrals and counted them up and showed much higher activity than appears to be showing on the performance data.... So that’s a major concern.’

Some examples were positive - the planning for the Wirral Memory Assessment Service drew on a shared PCT/acute trust data warehouse, along with electronic primary care data; and dementia services were modelled in detail (initially with the support of the Department of Health Care Services Efficiency Delivery programme). A more common story, though, was of a lack of accurate information about potential demand to inform service redesign:

*‘But this is the biggest handicap within the NHS*, *is accuracy and completeness of data. And be able to look at a number and go*, *‘That’s the number.’ And not look at six different numbers.’*

### Transaction costs

The scale and intensity of the commissioning work observed raised questions about whether it was proportionate to the likely impact on the quality and cost of patient care. Bartlett and Le Grand [7] argue that the transaction costs of running a quasi-market should be no higher than any cost savings generated by competition. As Williamson [3] noted, transaction costs may be *ex ante*, that is, incurred in the design and setting up of a contract, or *ex post*, that is, incurred in managing a contract once established.

A considerable proportion of our fieldwork examined the processes associated with *ex ante* transaction costs. In all three sites a considerable amount of labour went into the developmental aspects of commissioning, both the building of relationships, and technical tasks such as needs assessment, evidence review, demand mapping, writing a business case and service specification, contract negotiation, and preparation of a performance management framework. Across all three sites, the scale of these tasks combined with a limited supply of skilled commissioning staff meant that commissioners could only give their attention to a few areas of service provision at any one time.

Development work and associated *ex ante* costs were not exclusively the responsibility of commissioners within PCTs. For example, the remodelling of the Wirral Memory Assessment Service was largely driven by a psychiatrist working for a provider trust, while in Calderdale, staff from a provider trust did the work of arranging two large-scale workshops to plan new service models.

*Ex post* transaction costs were less obvious, mainly because the research focus was on services in transition (Additional file 1). However, within the three service developments – Wirral Memory Assessment Service, Somerset Diabetes Service, and Somerset Early Supported Discharge Service – which began operation during the study, we observed a blurring between *ex ante* and *ex post* transactional processes, over periods of a year or more. Monitoring of performance and activity levels fed into the development of a service specification and contractual arrangements. Constant review of service delivery helped to revise and refine what was expected of providers. While commissioners expected that the developmental work should reduce over time, there was not always a clear timetable for this; in the meantime, the cost was significant. Developing the Somerset Early Supported Discharge Service took the bulk of a PCT middle manager’s time for the year, with considerable input from senior colleagues. Monthly project meetings, bi-monthly care pathway groups, bi-monthly operational meetings, monthly contract meetings with each provider, plus one-off workshop and review events, all took up commissioners’ and providers’ time, as one commissioner observed:

‘*The work involved in writing up papers*, *doing the presentations*, *struggling with putting together a programme which just*, *you just couldn’t see*, *you know*, *even if you could get it off the ground*, *you couldn’t replicate it across the county.’*

In order to assess whether transaction costs are lower than any cost savings generated by competition, it is necessary to know what those cost savings are. However, an accurate picture of the cost of services was not always apparent, even in the three new services which were implemented during the fieldwork period. In all three cases, a more accurate picture of costs and potential cost savings was being constructed (supported by nationally driven mechanisms such as the splitting of payments for stroke care into smaller components, and a shift towards activity-based ‘Payment by Results’ in mental health care). However, in the meantime new service models were absorbed within large block contracts.

A final difficulty with assessing the cost/savings balance lay in unpicking what savings might be accruing, to whom, and when. For example the Somerset ESD service and Wirral Memory Assessment Service had the potential to generate long term savings in other health and social care provision, but were unlikely themselves to be cheaper to deliver than the services they replaced (in the case of WMAS, because of an increase in patient numbers, and in the case of ESD, because of the higher delivery costs of a peripatetic service). One provider reflected on where cost savings might accrue:

‘*If you take somebody with dementia*, *clearly they may well need more intensive acute services sooner than they would do*, *particularly because of their co-morbidities*, *more social care etc*, *so the actual saving isn’t going to be in [our] services necessarily because we are only a small part of wider services for people with dementia. It will be in other parts of the economy.’*

### Motivation

According to Bartlett and Le Grand [7], providers should be motivated, at least in part, by financial considerations, and purchasers by the concerns of users. Although they concede that the reality of the motivation on each side may be imperfect, underlying Bartlett and Le Grand’s analysis is the implicit assumption that the motivations and interests of purchaser and provider are distinct. However, in the six service areas, it was hard to discern such a distinction. Part of the reason was that money featured so little in the commissioning we were observing, perhaps because the financial aspects of commissioning were handled by separate teams from those dealing with developmental aspects of commissioning, and often behind closed doors, in negotiations described by one commissioner as ‘*protracted and painful’*.

Commissioners and providers appeared to share a motivation to improve patient care, which was reflected in all six areas in co-operative working in terms of service planning and development, based on positive relationships at organisational and individual levels. As one manager from a provider trust observed:

*‘I think we’ve always worked together.... [The commissioners] have always asked opinions*, *they’ve always sought out our view on service delivery*, *and they’ve never come across as being kind of punitive in their approach to us.’*

The same manager also described how the skills and knowledge of clinicians in particular were valued as a way to span the interests of providers and commissioners:

‘*People... say*, *“Oh*, *we can’t have providers in the room when we’re doing commissioning”. Well*, *of course you can and of course you should....So it’s very much a collaborative*, *inclusive process that then produces the model of service and also looks within that about affordability.*’

From the commissioners’ point of view, and to a lesser extent providers’, much of the motivation to bring about change came neither from financial concerns nor serving patients’ interests, but from national policy. Local commissioners were working to national guidance in terms of what they should be trying to achieve in long term conditions management [17,18] and wished to be seen to be performing well in these terms: delaying the onset and progression of long term conditions; finding cost savings and efficiencies; and reducing the need for unscheduled acute admissions. In addition, for each of the three long terms conditions, the Department of Health had produced a National Service Framework or Strategy [19-21], supported by NICE guidelines and by more detailed models of good practice. Such guidance was seen to shape the local performance management framework, as one commissioner described:

‘*We were keen that the outcome measures we looked at developing for the local CQUIN [a hospital payment for quality scheme]were things that reflected the outcome measures in the supplement of the National Dementia Strategy....So we’re very much looking at outcome measures that matter to people rather than just process measures.’*

### Cream-skimming

In a quasi-market, the use of health care by an individual should be related to need. For the quasi-market to work well, according to Bartlett and Le Grand [7], there should be no motivation for either providers or purchasers to ‘cream-skim’, that is, to pick and choose patients and exclude or limit those who are going to be more expensive to treat.

We found little that could be related directly to the question of ‘cream-skimming’, reflecting the generally small role played by money in the discussions we were observing. Instead, service planning discussions acknowledged the need to provide services for patients with different levels of need – particularly in relation to diabetes care, which followed a tiered model in all three sites. The difficulty of shifting patients between tiers of care was a constant theme, especially when it came to moving lower risk patients away from the care of hospital specialists. It is most unlikely that this represented cream-skimming; rather, a combination of professional reluctance to lose control and patient reluctance to change a familiar pattern of care.

### The difficulty of ‘parcelling’ care for long term conditions into neat units for quasi-market contracting purposes

Commissioning care in a quasi-market requires a boundary to be drawn around what is to be delivered in return for the money paid. Particular challenges arise when a pathway of care is to be delivered by multiple providers, as is frequently the case with services for people with long term conditions. The input required by patients is also likely to be open-ended and unpredictable.

The commissioning work we observed was largely concerned with the remodelling or re-provision of services. This meant that the necessary first step was to decide the scope and scale of the commissioning tasks to be undertaken, even before the boundaries were drawn around the provision of care. The units of work needed to be big enough to justify the labour of commissioning and associated *ex ante* transaction costs, whilst not being unwieldy.

In no case was there a ‘natural’ boundary around the service being commissioned. The diabetic podiatry service in Wirral lay at the intersection of diabetes care and podiatry – and so was shaped by considerations of budget, policy direction, staffing and management structures relating to each. The development of the Wirral Memory Assessment Service was tied to discussions on the broader strategy for dementia care, including work on medicines management and outcome indicators. In Somerset, although the ESD service itself was relatively clearly bounded (a time-limited intervention with specific objectives), it was perceived by both commissioners and providers as part of a bigger picture of provision for stroke care, with potential to have a positive impact on other areas of provision, as one clinician suggested:

‘*I think that the fact that*, *you know*, *consistently we’d got blockages within the acute Trusts that we hoped would be relieved by getting Early Supported Discharge’*

In Calderdale, there was an emphasis on ‘transformation’ of care services, with ambitious ideals for both dementia and diabetes care, but this had not yet moved to concrete commissioning developments.

## Discussion

In none of the six service areas were all of Bartlett and Le Grand’s [7] conditions for a well functioning quasi-market in health care in place. Choice of provider allowing competition to occur was generally absent or limited; information on demand and resource requirements was highly imperfect; motivations were complex; and transaction costs uncertain, but likely to be high. It was difficult to divide care into neat units for contracting purposes. As a result, it is scarcely surprising that the commissioning practice in relation to all six commissioning developments was dominated by a relational approach, largely based on trust and collaboration between commissioners and incumbent providers. In part, this may be explained by the persistence of relationships that pre-date the application of quasi-market principles to services for people with long term conditions. It also appears to be an adaptive response to the technical challenges of commissioning services for people with long term conditions. Both were first shown in the mid-1990s in the English NHS by Flynn et al (1996). Subsequent work on commissioner-provider relationships in the English NHS in the later 1990s and late 2000s has yielded similar findings [22-25] which resonate with those reported here.

Despite efforts from successive governments to introduce greater supplier competition and more ‘complete’ formal contracts into the system [22,23], non-market relationships remain strong. Existing relationships and loyalty play an important part in shaping practice, and were valued in the current study because the commissioners needed the knowledge (clinical and practical/operational) of providers in order to support them in their commissioning role. Hence, commissioners and providers remain, as Pestsoulas and colleagues put it, ‘mutually interdependent and contracting still relies heavily on relational networks and service norms’ ([23], p322). As a result, commissioners were wary of disrupting existing patterns of provision, and wary of conflict with providers, and there was very limited sign of commissioners threatening incumbent providers by proposing shifts in provision. Consistent with this, Petsoulas et al [23] show that providers and regulators still emphasise informal dispute resolution and negotiated cooperation even in the event of provider-commissioner conflicts. It seems that both parties respond paradoxically to pressure for more market-like behaviour with more socially embedded forms of contracting to maintain the functioning of the quasi-market in financially difficult times, a phenomenon predicted by Polanyi [26].

Although it can be argued on theoretical grounds that better constructed, legally enforceable contracts could help health care commissioners to exert greater influence with, and over, providers [27], the technical constraints faced in stimulating a market in this area and contracting for care, plus the costs of operating such a system, suggest that other factors are possibly more important [28]. While contracts can help to define the boundaries of a unit of provision (what Macneil [29] called *discreteness*), these boundaries were hard to pin down in the current study. Services remained intrinsically difficult to ‘commodify’ [30,31] (e.g. for competitive tendering).

The 'relational' aspects of commissioning and contracting, as compared with the 'transactional' element of formal contracting, appear to be ways of managing and to some extent overcoming these difficulties. Relational aspects include trust, common values, and networks, whether established or new [32-35]. The emphasis on relational contracting in our data may have been exaggerated by the fact that any use of the more transactional mechanisms to engender change seemed to take place elsewhere in the PCTs, outside the commissioning meetings associated with particular service areas (most likely in contract negotiations led by senior finance personnel and commissioning directors which the study was not able to observe). However, this divorcing of the more relational (service review, design and development) aspects of commissioning from the transactional aspects (contracting and performance monitoring) within the local practice of commissioning, is of note in itself since it calls into question how far the NHS quasi-market operates as a market in the way that policy makers may have intended, or whether elements from the ‘cycle of commissioning’ are applied in a manner that helps clinicians and managers to shape services for the future.

Our research focused exclusively on the commissioning of care for people with long-term conditions, so it is impossible to know whether all of the commissioning in the three PCTs functioned in the same manner. In services for people with long-term conditions, however, it appears that local managers and clinicians had developed and/or retained relational approaches to commissioning that draw together multiple providers to develop and plan new forms of more or less ‘integrated’ care.

International experience in planning and funding care for people with long-term conditions confirms the need to consider different approaches to contracting for acute and long term condition care which engender co-operation across organisations and services and reflect the way in which patients experience care. For example, there have been experiments in commissioning ‘chains of care’ in Sweden [36]. In New Zealand, experimental ‘alliance contracting’ shares risk and gains between all providers and commissioners, on a basis of trust and shared outcomes [37], while in the USA ‘accountable care organisations’[38] take a similar approach.

In England, there has been some experimentation with new forms of commissioning care for people with long-term conditions, as commissioners draw together a range of providers into a single contractual agreement where gains and risks are shared. Examples include the cardiovascular service for the people of Knowsley [39] and the Connected Care pilots supported by the charity Turning Point [40].

However, such approaches are challenging for NHS commissioners in that they run counter to the efforts of Government policy makers to encourage more market-like behaviour. Arguably, successive pro-market reforms of the NHS from the 1990s have tended to downplay or even ignore the possibility that services for people with long term conditions might need to be commissioned and provided differently from, for example, elective surgery [41].

In the three PCTs, conventional distinctions between the roles of commissioners and providers were often blurred as they worked together to redesign models of care. Clinicians and managers from provider organisations played a significant, and sometimes leading, role in needs identification, service planning and negotiating shifts of care between hospitals, primary care and community health providers. This behaviour is consistent with the official definition of ‘commissioning’ as opposed to ‘purchasing’ promoted by the Department of Health [8] which emphasises working closely with providers.

## Conclusions

This study reveals how and why the relational aspects of commissioning have remained predominant in the English NHS, at least in the local commissioning of care for people with long-term conditions. Our findings show the seemingly adaptive persistence of relational commissioning based on networks of interdependence and trust between commissioners and providers, and the difficulties faced by local commissioners in developing market relationships based on purchaser-provider contracting. This relational approach to commissioning challenges the simple notion of a quasi-market and can, instead, be seen as an understandable strategy of local commissioners faced with securing services that require the coordination of multiple providers, where skilled clinical staff are scarce and in the absence of good information on outputs, quality or costs.

## Competing interests

None.

## Authors' contributions

AP carried out data collection and analysis, and drafted large parts of the paper. NM contributed to analysis, identified the question and topic for the paper and drafted large parts of it. SS contributed to study design, data collection and analysis, and writing of the paper. RR contributed to study design and analysis, and commented on the paper. JS was the Principal Investigator for the study, and contributed to study design, data collection and analysis, and writing of the paper.

## Supplementary Material

Additional file 1 Commissioning activities observed in each of the six commissioning developments Click here for file

## Declarations

Publication of this supplement was funded by the Nuffield Trust.

This article has been published as part of *BMC Health Services Research* Volume 13 Supplement 1, 2013: The Limits of Market-based Reforms. The full contents of the supplement are available online at http://www.biomedcentral.com/bmchealthservres/supplements/13/S1.

## References

[B1] Hughes D Allen P Doheny S Petsoulas C Vincent-Jones P Co-operation and conflict under hard and soft contracting regimes: case studies from England and Wales BMC Health Serv Res 2013 13 Suppl 1 S7 23734604 10.1186/1472-6963-13-S1-S7 PMC3663652

[B2] Smith JA Shaw S Porter A Rosen R Blunt I Davies A Eastmure E Mays N Commissioning high quality care for people with long-term conditions Final report 2013 Southampton: NIHR Service Delivery and Organisation programme

[B3] Williamson OE Markets and hierarchies: analysis and antitrust implications 1975 New York: Free Press

[B4] Macneil IR Relational contract theory: challenges and queries Northwestern University Law Review 2000 94 877 907

[B5] Flynn R Williams G Pickard S Contracting for Health: quasi-markets and the National Health Service 1996 Buckingham: Open University Press

[B6] Allen P An economic analysis of the limits of market based reforms in the English NHS BMC Health Services Research 2013 13 Suppl 1 S1 23734962 10.1186/1472-6963-13-S1-S1 PMC3663645

[B7] Bartlett W Le Grand J Le Grand J and Bartlett W The Theory of Quasi-Markets Quasi-markets and Social Policy 1993 Basingstoke: Macmillan 13 34

[B8] Department of Health The NHS Contractors' Companion 2003 London: Department of Health

[B9] Øvretveit J Purchasing for health: a multi-disciplinary introduction to the theory and practice of purchasing 1995 Buckingham: Open University Press

[B10] Woodin J Walshe K, Smith JA Healthcare commissioning and contracting Healthcare Management 2006 1 Maidenhead: Open University Press

[B11] Mays N Hand K A Review of options for health and disability support purchasing in New Zealand Treasury Working Paper 00/20 2000 Wellington: New Zealand Treasury

[B12] Department of Health Health Reform in England: Update and next steps 2005 London: Department of Health http://webarchive.nationalarchives.gov.uk/20130107105354/http://www.dh.gov.uk/prod_consum_dh/groups/dh_digitalassets/@dh/@en/documents/digitalasset/dh_4124727.pdf (accessed on 10 May 2011)

[B13] Department of Health World Class Commissioning: competencies 2007 London: Department of Health http://webarchive.nationalarchives.gov.uk/20130107105354/http://www.dh.gov.uk/prod_consum_dh/groups/dh_digitalassets/@dh/@en/documents/digitalasset/dh_080964.pdf (accessed on 8 January 2013)

[B14] Department of Health Equity and excellence; liberating the NHS Cm 7881 2010 London: The Stationery Office

[B15] Smith J Charlesworth A NHS reforms in England: managing the transition 2011 London: Nuffield Trust

[B16] Hammersley M Gomm R Foster P Gomm R, Hammersley M, Foster P Case study and theory Case Study Method 2000 London: Sage 237 258

[B17] Department of Health The National Service Framework for Long Term Conditions 2005 London: Department of Health

[B18] Patel KCR Spilsbury P The UK National Health Service approach to the economic crisis Journal of the Royal Society of Medicine 2010 103 4 123 124 10.1258/jrsm.2010.100061 20382897 PMC2853410

[B19] Department of Health National Service Framework for Diabetes 2001 London: Department of Health

[B20] Department of Health National Service Framework for Stroke 2007 London: Department of Health

[B21] Department of Health National Dementia Strategy 2007 London: Department of Health

[B22] Allen P A socio-legal and economic analysis of contracting in the NHS internal market using a case study of contracting for district nursing Social Science & Medicine 2002 54 255 66 10.1016/S0277-9536(01)00025-9 11824930

[B23] Petsoulas C Allen P Hughes D Vincent-Jones P Roberts J The use of standard contracts in the English National Health Service: a case study analysis Social Science & Medicine 2011 73 2 185 92 10.1016/j.socscimed.2011.05.021 21684643

[B24] Hughes D Petsoulas C Allen P Doheny S Vincent-Jones P Contracts in the English NHS: market levers and social embeddedness Health Sociology Review 2011 20 3 321 37 10.5172/hesr.2011.20.3.321

[B25] Checkland K Snow S McDermott I Harrison S Coleman A Management practice in Primary Care Trusts: the role of middle managers Final report 2011 NIHR Service Delivery and Organisation Programme

[B26] Polanyi M The great transformation: the political and economic origins of our time 1944 Boston: Beacon Press

[B27] Ferlie E McGivern G Relationships between health care organisations: a critical overview of the literature and a research agenda 2003 London: National Co-ordinating Centre for NHS Service Delivery and Organisation R&D Programme

[B28] Smith J Woodin J Walshe K, Smith JA Purchasing healthcare Healthcare management 2011 2 Maidenhead: Open University Press

[B29] Macneil IR Values in contract: internal and external Northwestern University Law Review 1983 78 340 418

[B30] Appardurai A The social life of things: commodities in a cultural perspective 1986 Cambridge: Cambridge University Press

[B31] Marx K Engels F The communist manifesto (1848) Trans. Samuel Moore 1910 1888 Chicago: Charles H Kerr & Co

[B32] Ferlie E The new public management in action 1996 Oxford: Oxford University Press

[B33] Walsh K Public services and market mechanisms: competition, contracting and the new public management 1995 Basingstoke: Macmillan

[B34] Lapsley I Llewellyn S Flynn R, Williams G Statements of mutual faith: soft contracts in social care Contracting for health: quasi-markets and the National Health Service 1997 Maidenhead: Open University Press

[B35] Shaw SE Ashcroft J Petchey RP Developing sustainable relationships for health improvement: the case of public health and primary care in the UK Critical Public Health 2006 16 2 127 35

[B36] Åhgren B Chain of care development in Sweden: results of a national study International Journal of Integrated Care 2003 3 e01 16896423 PMC1483939

[B37] McCormick I Hooton R The ‘ABC’ and ‘123’ of alliances in health’ New Zealand Doctor 2011

[B38] Fisher E Staiger D Bynum J Gottlieb D Creating accountable care organizations: the extended hospital medical staff Health Affairs 2007 26 1 44 57 10.1377/hlthaff.26.1.w44 17148490 PMC2131738

[B39] Ham C Smith J Eastmure E Commissioning integrated care in a liberated NHS 2011 London: The Nuffield Trust

[B40] Turning Point Connected Care impact report 2012 London: Turning Point

[B41] Millar R Powell M Dixon A What was the programme theory of New Labour’s Health System Reforms? Journal of Health Services Research & Policy 2012 17 Suppl 1 7 15 22315474 10.1258/jhsrp.2011.010189

