# Supplementary material for: Commissioning healthcare for people with long term conditions: the persistence of relational contracting in England’s NHS quasi-market
Source: BMC Health Serv Res. 2013 May 24;13(Suppl 1):S2. doi: 10.1186/1472-6963-13-S1-S2 (PMC3663656; doi:10.1186/1472-6963-13-S1-S2)
Supplement: Additional file 1 — Commissioning activities observed in each of the six commissioning developments [file 1472-6963-13-S1-S2-S1.docx]

**Commissioning activities observed in each of the six commissioning developments**

| **Commissioning development** | **Commissioning activity being tracked** | **Key features observed** | **Stage of development during fieldwork period** | **Providers** |
| --- | --- | --- | --- | --- |
| Calderdale – a strategic plan for diabetes services | Review of existing provision of diabetes care and discussion of plans for strategic remodelling | - Difficulty of achieving consensus on what to do - Good performance in terms of clinical outcomes and costs provided meant that there was little challenge to ‘traditional’ model of care | Discussion and planning | GPs  Community health  Acute trust |
| Calderdale – transformation of dementia services | A strategic review of all dementia care, including social care and third sector provision as well as health care. The emphasis was on early intervention and supporting independence. | - The review process led by the local partnership (mental health) trust, with involvement from local stakeholders - Part of a wider programme of transformational change across an area covered by 3 PCTs. - An ambitious timetable for change, but ideas not translated into service changes during the fieldwork period. | Discussion and planning | Partnership trust  GPs  Partnership trust  Acute trusts x2  Third sector providers |
| Somerset - Diabetes Service | Remodelling of diabetes care into a three tier service, with intermediate care delivered by nurses in community clinics. | - A shift of care and staff from acute providers to community settings, with an increasing role for diabetes specialist nurses. - Restructuring driven by the PCT over three years, dogged project management, significant clinical and staff engagement, and continuing involvement in implementation and review. | Commenced operation | Acute trusts x4  Community health provider (transitioned from PCT to partnership trust during fieldwork period)  GPs  Third sector |
| Somerset – Early Supported Discharge (ESD) Service for Stroke | The setting up of a service delivering intensive therapy at home to patients recovering from a stroke | - A relatively small scale, intensive service, following a national model. - Strong drive to bring patient numbers up to a target – but a struggle to identify enough suitable people. | Commenced operation | Acute trusts x4  Community health provider (transitioned from PCT to Partnership trust during fieldwork period) |
| Wirral – diabetic podiatry service | Review of diabetic podiatry to resolve operational problems (including records management and referral practice between community and acute tiers). | - Inconsistencies in data mean that it was not yet possible to identify patient numbers and costs at each level. - Continuing challenges around ensuring appropriate transition between the three tiers of service delivery. | Discussion, planning, modelling | GPs  Community health provider  Acute trusts |
| Wirral - Memory Assessment Service | Establishment of a new community based service for diagnosis and treatment of dementia in community based clinics led by nurses with support from psychiatrists. | - This service succeeded a previous model delivered by specialist GPs. - Activity levels exceeded all expectations within months. | Commenced operation | Partnership Trust  Third sector provider |
